# Cellular and Molecular Effect of MEHP Involving *LXRα* in Human Fetal Testis and Ovary

**DOI:** 10.1371/journal.pone.0048266

**Published:** 2012-10-30

**Authors:** Vincent Muczynski, Charlotte Lecureuil, Sébastien Messiaen, Marie-Justine Guerquin, Thierry N’Tumba-Byn, Delphine Moison, Wassim Hodroj, Hinde Benjelloun, Jan Baijer, Gabriel Livera, René Frydman, Alexandra Benachi, René Habert, Virginie Rouiller-Fabre

**Affiliations:** 1 University Paris Diderot, Sorbonne Paris Cité, Laboratory of Development of the Gonads, Unit of Stem Cells and Radiation, Fontenay-aux-Roses, France; 2 CEA, DSV, iRCM, SCSR, LDG, 92265 Fontenay-aux-Roses, France; 3 INSERM, Unité 967, F-92265, Fontenay aux Roses, France; 4 Flow Cytometry Facility, CEA – DSV/iRCM/SCSR, F-92265 Fontenay aux Roses, France; 5 Service de Gynécologie-Obstétrique, Hôpital A. Béclère,- Université Paris Sud, F-92141 Clamart, France; National Cancer Institute, United States of America

## Abstract

**Background:**

Phthalates have been shown to have reprotoxic effects in rodents and human during fetal life. Previous studies indicate that some members of the nuclear receptor (NR) superfamilly potentially mediate phthalate effects. This study aimed to assess if expression of these nuclear receptors are modulated in the response to MEHP exposure on the human fetal gonads *in vitro*.

**Methodology/Principal Findings:**

Testes and ovaries from 7 to 12 gestational weeks human fetuses were exposed to 10^−4^M MEHP for 72 h *in vitro.* Transcriptional level of NRs and of downstream genes was then investigated using TLDA (TaqMan Low Density Array) and qPCR approaches. To determine whether somatic or germ cells of the testis are involved in the response to MEHP exposure, we developed a highly efficient cytometric germ cell sorting approach. *In vitro* exposure of fetal testes and ovaries to MEHP up-regulated the expression of *LXRα*, *SREBP* members and of downstream genes involved in the lipid and cholesterol synthesis in the whole gonad. In sorted testicular cells, this effect is only observable in somatic cells but not in the gonocytes. Moreover, the germ cell loss induced by MEHP exposure, that we previously described, is restricted to the male gonad as oogonia density is not affected *in vitro*.

**Conclusions/Significance:**

We evidenced for the first time that phthalate increases the levels of mRNA for *LXRα*, and *SREBP* members potentially deregulating lipids/cholesterol synthesis in human fetal gonads. Interestingly, this novel effect is observable in both male and female whereas the germ cell apoptosis is restricted to the male gonad. Furthermore, we presented here a novel and potentially very useful flow cytometric cell sorting method to analyse molecular changes in germ cells versus somatic cells.

## Introduction

Phthalic esters are compounds widely used as plasticizers. About 3 million tons of phthalic esters per year are produced world-wide and they can be found in many everyday products, such as PVCs, plastic bags, food packaging, cosmetics, industrial paints as well as blood transfusion packs [1], [2], [3]. Due to the non-covalent nature of their link with plastics, phthalates can easily leach from these products and, consequently, be ingested [4]. This is particularly worrying in pregnant women, since phthalates can reach the fetus. Indeed, the average level of Mono-EthylHexyl Phthalate (MEHP), the main active metabolite of Di-EthylHexyl Phthalate (DEHP), in cord blood is about 2×10^−6^ M [5], [6] with maximal concentrations up to 10^−5^ M [5].

**Figure 1:**
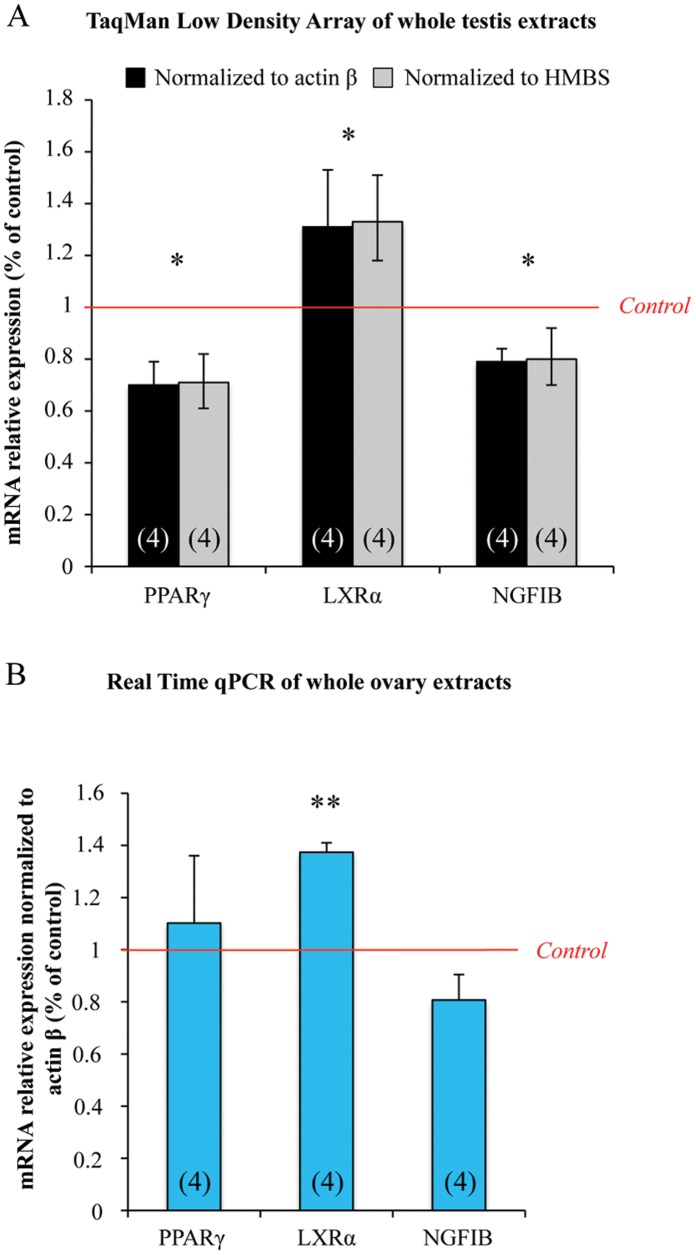
MEHP exposure affects the expression of nuclear receptors in human fetal testes and ovaries. Testes and ovaries from 7 to 12 week-old human fetuses were cultured with or without 10^−4^ M MEHP for 3 days and then mRNAs were isolated from whole gonad. (A), NRs superfamily TLDA plates were run with whole testis samples. Results were normalized to *actin β* or *HMBS* expression and the 3 differentially expressed NRs are shown as fold changes relative to the control values. Histograms represent the mean ± standard error of the results using 4 independent fetal testis cultures. (B), Transcriptional level of *PPARγ*, *LXRα* and *NR4A1* was analyzed by real-time qPCR in fetal ovaries. Results were normalized to *Actin β* expression and are shown as fold changes relative to control values. Histograms represent the mean ± SEM of 4 different ovaries from different fetuses (as indicated in the respective column). *p<0.05, **p<0.01 in paired t-tests recommended when comparing few samples.

The toxic effects of phthalates on the development of the reproductive organs/functions have been widely described in rodent species. In the rat, *in utero* exposure (by force-feeding pregnant rats) to various phthalate ester subtypes has deleterious effects on testosterone production associated with abnormal aggregation of fetal Leydig cells [7], [8], [9], [10], [11], [12]. These data have been confirmed also by using the *in vitro* method of organotypic culture of rat fetal gonads [13]. However, in the mouse, the effect of MEHP on testosterone secretion/production varies depending on the experimental conditions and the age of the testis explants [14]. Conversely, in the marmoset (*Callithrix jacchus*) no effects were observed following *in vivo* exposure to phthalates [15]. We have previously demonstrated that 72 h exposure to MEHP of the human fetal testis in culture has no effect on testosterone production or Leydig cell aggregation, but induces rapid germ cell loss in a dose-dependent (10^−4^ M and 10^−5^ M) manner [16], [17]. In the same way, steroidogenesis is not affected by DBP exposure in human fetal testis xenograft model [18]. If the deleterious effect of phthalates on fetal Leydig cells seems to be restricted to the rat, the disruption of germ cell development during fetal life has been reported not only in human [16], [17] but also in rat and mouse fetal testes both *in vivo* [19], [20], [21], [22] and *in vitro* [13], [14], [23], [24].

**Figure 2:**
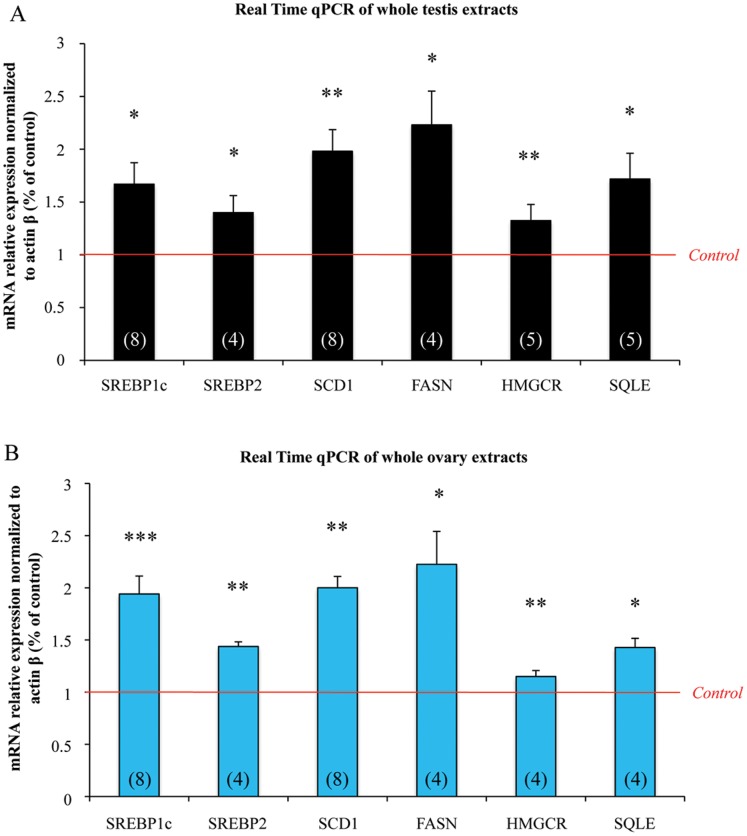
In human fetal gonads, exposure to MEHP significantly increases the mRNA expression of LXRα downstream genes involved in the cholesterol and lipid pathways. Ovaries and testes from 7 to 12 week/old human fetuses were cultured with or without 10^−4^ M MEHP for three days. mRNAs were isolated from whole gonads and the transcriptional level of LXRα downstream genes was analyzed by real-time qPCR in testes (A) and ovaries (B). Results were normalized to *Actin β* expression and are shown as fold changes relative to control values. Histograms represent the mean ± SEM of 4 to 8 different ovaries/testes from different fetuses (as indicated in the respective column). *p<0.05, **p<0.01, ***p<0.001 in paired t-tests recommended when comparing few samples.

Beyond their well-described reprotoxic effects, phthalates are also suspected to interact with different members of the Nuclear Receptor (NR) superfamilly or to act via their pathways by modulating expression of some nuclear receptors and their targets in various organ models. Recent studies have demonstrated that CAR (Constitutive Androstane Receptor) and its target genes are activated following DEHP exposure in mice and human hepatocytes [25]. Transcription of Pregnane X receptor (PXR) target genes is also up-regulated upon MEHP exposure in a human hepatocarcinoma cell line [26]. Peroxysome-Proliferator Activated Receptor (PPAR) activation has been widely linked to the adverse effects of exposure to phthalates in adult mice [27]. Ward and collaborators reported that genetic ablation of PPARα suppresses the hepatotoxic effect of DEHP in adult mice [28]. Moreover, models of *in vitro* transactivation assay demonstrated that MEHP is able to activate human and rodent PPARα and PPARγ isoforms [29]. However, in PPARα knock-out mice, the deleterious effects of DEHP exposure on male genitalia were only partially blocked, suggesting that some toxicological effects of phthalate esters may be mediated also by other NRs [26], [28]. In the rat or mouse fetal and neonatal testes, the expression of different NRs is also modulated in response to *in vivo* phthalate exposure. Indeed, for *PPARγ*, both mRNA and protein expression are down-regulated [30], [31]. Transcriptomic analyses also reported that *NGFIB* (NR4A1) and *NOR1* (NR4A3), two orphan members of the NR superfamily, are up-regulated following phthalate exposure [21], [32], [33]. Furthermore, species-differences have been described. For instance, DEHP exposure induces PPARα target genes in mice and rats, but not in marmosets [34]. Similarly, PPARα-humanized mice have a different phenotypic response to DEHP treatment compared to wild type mice [35]. It is therefore essential to identify which NRs and downstream signaling pathways are modulated following exposure to phthalates in human fetal gonads.

**Figure 3:**
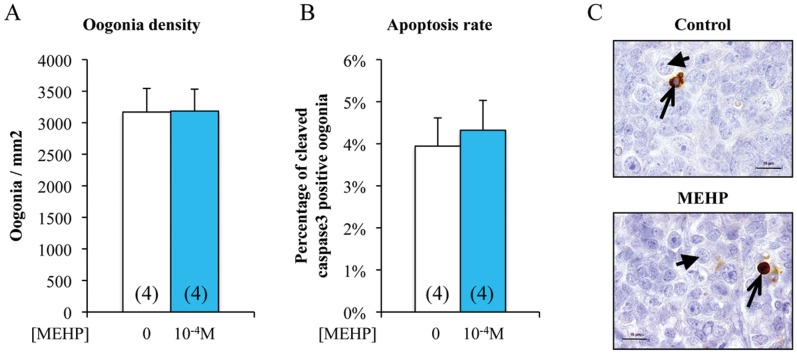
*In vitro* exposure to MEHP does not affect the number or apoptosis rate of germ cells in human fetal ovaries. Ovaries from 7 to 12 week/old human embryos were cultured with or without 10^−4^ M MEHP for three days. At the end of the culture period, they were fixed with Bouin’s solution and stained with hematoxylin and eosin. Oogonia density (number of germ cells per mm^2^ tissue) was measured based on the morphological analysis (A) and germ cell apoptosis rate (B) based on the expression of cleaved Caspase-3 (brown) (C). Histograms represent the mean ± SEM of four different ovaries from different fetuses (as indicated in the columns). Arrowheads indicate oogonia and arrows cleaved Caspase- 3 positive oogonia. Bar, 15 µm.

In this study, we analyzed the transcriptional regulation of the different members of the NR superfamily and their downstream pathways in human male and female fetal gonads following exposure to MEHP in organotypic cultures. Since we previously showed that fetal germ cells are particularly affected by MEHP exposure in the human male gonad [16], we also developed a highly efficient cytometric cell sorting approach to determine in which cell population of the testis (somatic or germ cells) MEHP acts.

## Materials and Methods

### Ethic Statement

This study was approved by the Antoine Béclère Hospital Ethics Committee as well as the French Agence de Biomedecine under the reference number PFS12-002. All women gave their written consent on a signed document stipulating that a scientific program will be carried out on their fetus.

### Recovery of Human Fetal Gonads

Human fetal gonads were obtained from pregnant women referred to the Department of Obstetrics and Gynecology at the Antoine Béclère Hospital, Clamart (France) for legally induced abortion in the first trimester of pregnancy (*i.e.* from the 7^th^ until the 12^th^ week post conception) as previously described [36], [37]. We found gonads within the abortive material in approximately 50% to 60% of cases.

**Figure 4:**
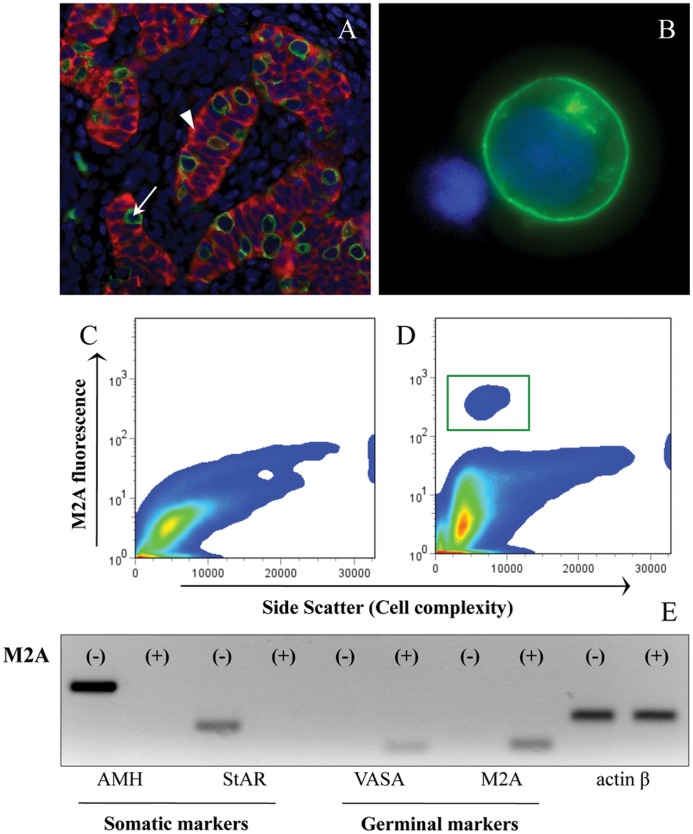
Isolation of M2A-positive and –negative cells from human fetal testes by flow cytometric cell sorting. Histological immunofluorescent staining for M2A (green) and AMH (red) in human fetal testis (A). M2A staining in living dissociated testicular cells (B). DNA was stained with DAPI (blue color in (A) and (B)). Cell sorting profiles of M2A fluorescence in fetal testicular cells incubated with isotype control (C) or anti-M2A antibody (D). The green square (D) represents the cell fraction sorted as M2A-positive. mRNA expression of different cell type markers in M2A-positive and -negative cell fractions (E). *AMH* and *StAR* are markers of Sertoli and Leydig cells, respectively, while *VASA* and *M2A* characterize the germ cell population. *Actin β* was used as control. White arrow indicates M2A positive cell, white arrowhead indicates AMH positive cell.

### Organ Cultures

Human fetal gonads were cultured on Millicell-CM Biopore membranes (pore size 0.4 µm, Millipore, Billerica, MA) as previously described [16], [37]. The culture medium was phenol red-free Dulbecco modified Eagle medium/Ham F12 (1∶1) (Gibco, Grand Island, NY) supplemented with 80 µg/mL gentamicin (Sigma, St. Louis, MO). Each human gonad (ovary or testis) was cut into small pieces and three pieces from the same gonad were placed on a membrane floating on 320 µL of culture medium in tissue culture dishes and cultured at 37°C in a humidified atmosphere containing 95% air and 5% CO2 [16], [38]. The response to MEHP (TCI Europe; Antwerp, Belgium) was investigated by comparing explants from the same gonad cultured 4 days with the 3 last days in medium containing the solvent (DMSO, 1∶4000, control) or MEHP (10^−4^ M). This dose was selected as we previously demonstrated that it produces strong deleterious effects without inducing toxicity in the testis [16]. Culture medium was changed every 24 h. At the end of the culture period, all explants from the same gonad were placed in RLT buffer for RNA isolation with Qiagen kits or in collagenase/DNAse solution for cell sorting. In addition, some fetal ovary explants were fixed in Bouin’s solution for 1 hour, dehydrated and embedded in paraffin.

**Figure 5:**
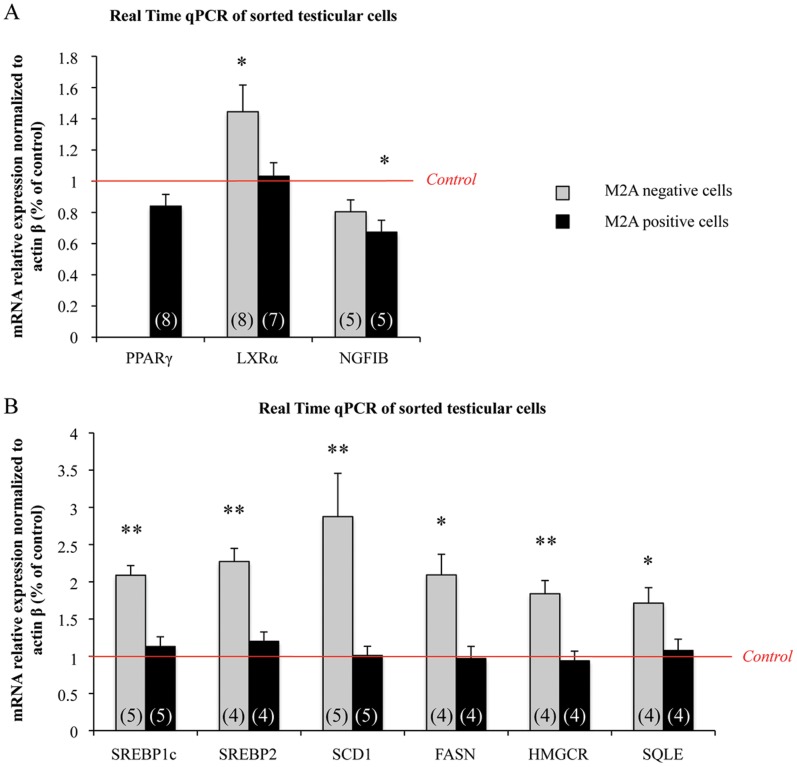
mRNA expression of *PPARγ*, *LXRα* and *NR4A1* as well as of LXRα downstream genes in sorted germinal and somatic cells from fetal testes. Human fetal testes were treated, or not, with 10^−4^ M MEHP for 3 days in organotypic cultures. Testes were then dissociated and the M2A-positive and -negative cell fractions were sorted. The relative mRNA expression of *PPARγ*, *LXRα* and *NR4A1* (A) as well as of LXRα downstream genes involved in cholesterol and lipid synthesis (B) was quantified in the M2A-positive and -negative cell populations by real-time qPCR. Results were normalized to *actin β* expression and are shown as fold changes relative to controls. The black columns represent M2A-positive cells and the grey columns the M2A-negative cells. The histograms are the mean ± SEM of 4 to 8 different testis from different fetuses (as indicated in the respective column). *p<0.05, **p<0.01 in paired t-tests as recommended when comparing few samples.

### Fetal Germ Cell Sorting

#### Testicular cell dissociation

At the end of the culture period, all the explants from the same testis were pooled and placed in PBS supplemented with 1% collagenase and 10% DNase. Cells were enzymatically dissociated under gentle shaking at 37.5°C for 5 min followed by repeated pipetting. Cells were then centrifuged (1200 rpm for 7 min) to remove the dissociation solution and resuspended in blocking solution (PBS/5% BSA) for 20 min.

**Figure 6:**
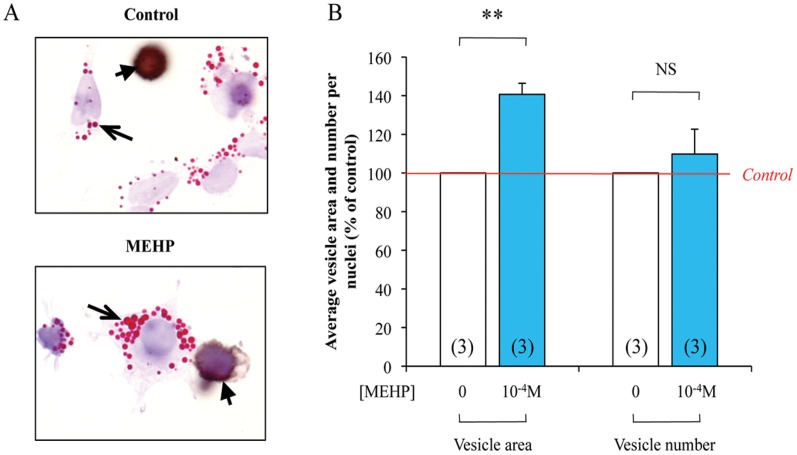
Quantification of lipid vesicle in dispersed testis cells following 72 **h of 10^−4^** **M MEHP exposure.** Human fetal testes were treated, or not, with 10^−4^ M MEHP for 3 days in organotypic cultures. Testes were then dissociated and Oil Red O (Red, arrows) and M2A (Brown, arrowheads) staining were performed (A). In the somatic cells, quantification of lipid vesicle number per nuclei and average vesicle area was performed using Image J software (B). Control are set to 100% and treated are expressed in percentage of the control. The histograms are the mean ± SEM of 3 independent experiments from different fetuses (as indicated in the respective column). NS  =  Not Significant, **p<0.01 in paired t-tests as recommended when comparing few samples.

#### M2A staining

Dissociated cells were centrifuged to discard the supernatant and incubated with a mouse anti-human M2A monoclonal antibody (1∶200 Ab77854, Abcam) at room temperature for 40 min. After three washes in PBS, cells were incubated with an Alexa-488 coupled rabbit anti-mouse antiserum (1∶500, Invitrogen) for 20 min, then briefly washed and suspended in PBS with 2% BSA for cell sorting.

#### Cell sorting

Sorting was performed using a BD-Influx™ biohazard system (BD Biosciences; San Jose, CA; USA). 2.5 ng/ml Propidium Iodide (Sigma-Aldrich) was added to each sample to exclude dead cells. Alexa-488 fluorescence was excited at 488 nm (200 mW) and fluorescent signals collected through a 520/40 bandpass filter. Two populations were identified and sorted: an M2A-negative fraction (i.e., testicular somatic cells) and an M2A-positive fraction (germ cells). Cells were sorted directly in RLT buffer for RNA extraction and expression analyses.

**Figure 7:**
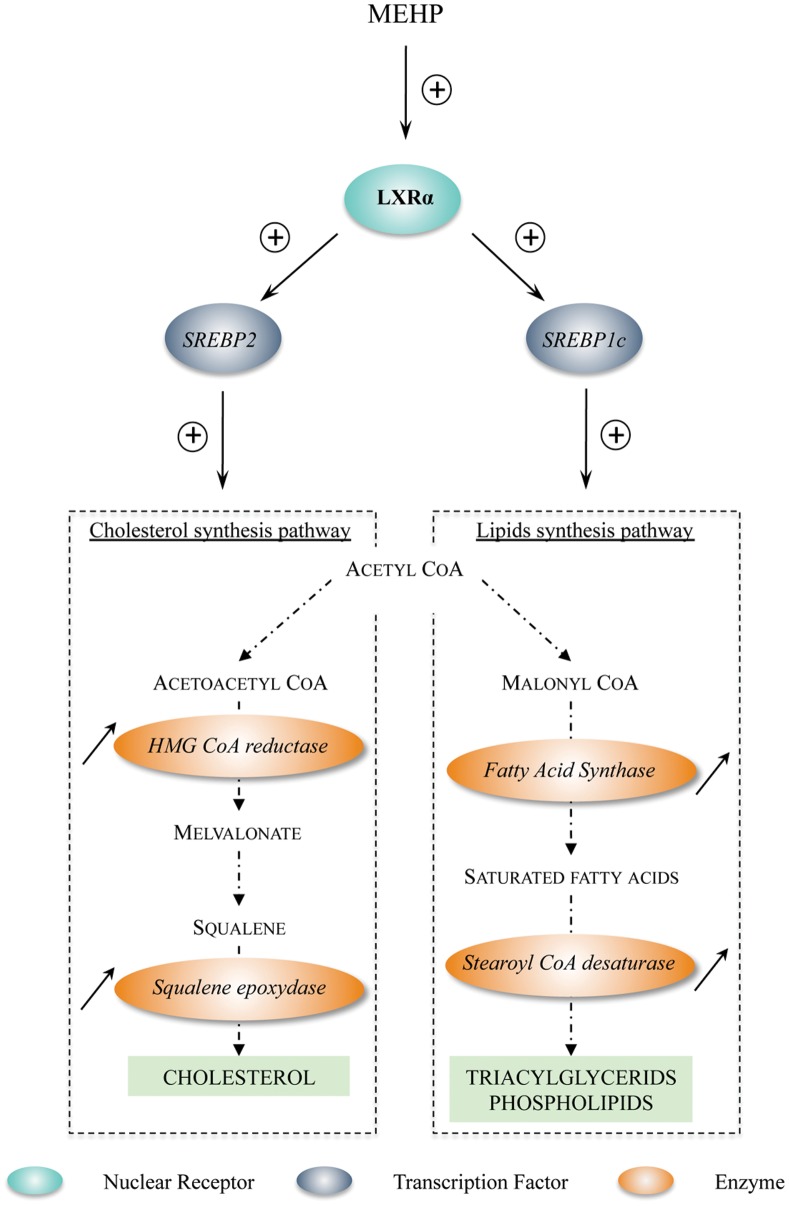
Working model of LXRα transcriptional up-regulation induced by MEHP exposure and effects on downstream genes involved in cholesterol/lipid synthesis. First, exposure to MEHP up-regulates *LXRα* expression. Increased LXRα activity then stimulates the mRNA expression of *SREBP1c* and *SREBP2* that positively regulate, respectively, the transcriptional level of lipid and cholesterol synthesis enzymes, therefore potentially leading to an increase in cholesterol and lipid synthesis in cells. Adapted from [59].

### Quantitative Real-time Polymerase Chain Reaction (qPCR)

Total RNA was extracted using either the RNeasy Mini Kit (Qiagen) (ovary and testis explants) or the RNeasy Micro Kit (sorted testicular cells), both containing a DNase I digestion step to avoid genomic DNA contamination. RNAs were quantified using a NanoDrop ND-100 spectrophotometer (Labetch, France). Each sample was then reverse transcribed by using the High Capacity cDNA Reverse Transcription kit (Applied Biosystems, Courtabeuf, France). qPCR was carried out as previously described [16] using a 7900HT Fast Real-Time PCR system (Applied Biosystems). Each sample was run in duplicate and negative controls (without samples) were run for each primer combination. Every gene of interest was first standardized to the reference gene, and then the treated condition was represented in relative expression of the control. Primers are listed in Table S1.

### TaqMan Low Density Array (TLDA)

TLDA plates (Applied Biosystems) designed for assessing the expression of the 48 known human NRs and 16 housekeeping genes were used. The geNorm software was used to determine the most appropriate control genes for our experimental conditions [39]. HydroxyMethylBilane Synthase *(HMBS)* and Actin beta *(Actin β)* were found to be the two more stable genes after MEHP treatment. For these experiments, 500 ng RNA were required for reverse transcription with the High Capacity cDNA Reverse Transcription kit. The experiment was then performed according to manufacturer’s instructions using the 7900HT Fast Real-Time PCR system (Applied Biosystems). Each sample was run in triplicate.

### Histological Analyses

#### Female germ cell counting

Serial gonad sections were deparaffinized, rehydrated and stained with hematoxylin and eosin to count oogonia. The germ cell density was calculated by dividing the germ cell number by the area of the counted section as previously described [40]. All counts and measurements were carried out blind to the treatment and using the Histolab analysis software (Microvision Instruments, Evry, France).

#### Immunohistochemical analysis of cleaved Caspase-3 expression

Because caspase­3 is involved in most apoptotic pathways [41], we used its immuno­ detection to quantify the rate of apoptosis. The primary anti-Cleaved Caspase-3 Asp 175 antibody (1∶100, Cell Signaling, Beverly, MA) was detected using biotinylated goat anti-rabbit secondary antibodies in 5% Normal Goat Serum (NGS) and the avidin-biotin-peroxidase complex (Vectastain Elite ABC kit, Vector Laboratories, Burlingame, CA). Peroxidase activity was visualized using 3,3′-diaminobenzidine (DAB) as substrate. For all immunohistochemical staining, negative controls were done by omitting the primary antibody.

### Oil Red O Staining

At the end of the culture, testicular cells were dispersed as described (section *Fetal germ cell sorting/Testicular cell dissociation*). Dispersed cells were then resuspended in DMEM in 8 wells Labteck culture plates (Nunc, Denmark) and placed at 37°C for 5 h. The cells were fixed with 4% formaldehyde during 10 min at room temperature. Cells were further stained using classical Oil Red O staining protocol, which shows the presence of triglyceride deposits. M2A immunological staining was performed as previously described (section *Fetal germ cell sorting/M2A staining*) to visualize germ cells. Lipid vesicle number per nuclei and average area in the somatic cells were quantified using Image J software (Freeware under the general public license) with the particle detection plug-in.

### Statistical Analysis

All values are expressed as mean ± SEM. For all analyses, the significance of the differences between paired values of treated and untreated gonads was evaluated using the paired t-test as recommended for small-size samples.

## Results

### MEHP Modulates the mRNA Level of Members of the Nuclear Receptor Superfamily in Human Fetal Male and Female Gonads

To determine whether members of the nuclear receptor (NR) superfamily are transcriptionally modulated by phthalates in human fetal testis, we analyzed the mRNA expression of the 48 known human NRs using TLDA plates and mRNA from gonads cultured in control condition (DMSO) and after exposure to MEHP. In control condition, 41 of the 48 NRs where detected in each first trimester human fetal testis sample (Table S2).

Following *in vitro* exposure to 10^−4^ M MEHP for three days, changes in mRNA levels were found only for *PPARγ* (NR1C3), *NGFIB* (NR4A1) and *LXRα* (NR1H3) (Figure 1A) and all the other 38 detected RNs were not modified (Table S2). The mRNA level of *PPARγ* and *NGFIB* was significantly reduced by 30% and 20% respectively, whereas *LXRα* showed a 30% increase (Figure 1A).

To test if this transcriptional modulation is specific to the male gonad, we assessed whether *LXRα, PPARγ* and *NGFIB* mRNA levels could also be affected by MEHP exposure in the human fetal ovary. Similarly to what observed in the testis, *LXRα* mRNA level was increased by 40% in treated ovaries in comparison to control samples (Figure 1B). Conversely, the transcriptional level of *PPARγ* and *NGFIB* in ovaries was not modified by MEHP exposure (Figure 1B).

### MEHP Exposure Modulates Downstream Genes of LXRα Both in Male and Female Gonads

As LXRα is the only NRs modulated similarly in fetal testis and ovary, we choose to analyze effect of MEHP on its downstream genes. LXRα is mainly a regulator of lipid and cholesterol homeostasis, we then investigated the effect of MEHP exposure on the expression of different transcription factors and enzymes involved in these two pathways using a qPCR approach. After a 72 h of 10^−4^ M MEHP exposure, genes involved in lipid metabolism, such as Sterol Regulatory Element-Binding Protein 1c (*SREBP1c*), which regulates the fatty acid pathways, and the downstream enzymes Stearoyl-CoA Desaturase 1 (*SCD1*) and Fatty-Acid Synthase (*FASN*) were up-regulated in human fetal testis (Figure 2A). Similarly, the expression of genes involved in cholesterol synthesis, such as Sterol Regulatory Element-Binding Protein 2 (*SREBP2*), 3-Hydroxy-3-MethylGlutaryl-CoA Reductase (*HMGCR*) and Squalene Epoxydase (*SQLE*), was increased by 1.4, 1.3 and 1.7 times respectively compared to the control (Figure 2A). Similar findings were observed as soon as 24 h of exposure (Data not shown).

In the same way, fetal ovaries presented an increased mRNA level of *SERBP1c* and *SREBP2* and of the downstreamm enzymes *SCD1*, *FASN*, *HMGCR* and *SQLE* (by 1.2 to 2.2 times in comparison to controls) after 72 h of 10^−4^ M MEHP exposure (Figure 2B).

### MEHP does not Affect Ovarian Germ Cell Development during the First Trimester of Pregnancy

We observed similar MEHP effects on transcriptional regulation of LXRα and downstream genes in both human fetal testis and ovary. As in the male gonad, MEHP exposure induces germ cell apoptosis leading to strong germ cell loss [16], we exposed fetal ovary explants to 10^−4^ M MEHP for three days and then assessed oogonia apoptosis. This exposure did not modify either the oogonia density (number of germ cells per mm^2^ of tissue) (Figure 3A) or the rate of female germ cell apoptosis (4%) as determined by counting the number of cleaved Caspase 3 positive germ cells (Figure 3 B and C).

### Use of M2A Staining in Cytometric Cell Sorting is a New Method to Isolate Human Gonocytes

As in the human testis, germ loss is a well-described effect of MEHP, we decided to identify which testicular cells are concerned by *LXRα* and downstream genes up-regulation. In this purpose, we developed a novel flow cytometric cell sorting approach based on the staining of M2A, a trans-membrane antigen that is expressed in 90 to 95% of germ cells during the first trimester of pregnancy [42].

First, using an immunohistological approach, we observed that M2A staining (green) was exclusively localized in the gonocytes characterized as large seminiferous cells that do not express anti-Müllerian hormone (AMH: a marker of Sertoli cells, in red), (Figure 4A). Dissociated testicular cells showed a population of large cells that were strongly M2A positive (green) with essentially no background staining in other cells (Figure 4B).

Secondly, the bivariate histogram of the Alexa-488 fluorescence (M2A) versus the side scatter (SSC) showed that the M2A-positive fraction was well separated from the M2A-negative population and therefore both populations were easily retrievable by cell sorting (Figures 4C and 4D). The purity of both populations was confirmed using RT-qPCR. Indeed, the M2A-positive fraction expressed only germ cell markers (*M2A* and *VASA*), whereas the M2A-negative fraction expressed exclusively Sertoli cell (*AMH*) and Leydig cell (Steroidogenic Acute Regulatory protein, *StAR*) markers (Figure 4E). This demonstrates that the M2A-positive and -negative cell populations are almost pure and that gonocytes are exclusively found in the M2A-positive fraction.

### MEHP Modulates NRs Expression and LXRα Downstream Genes in Sorted Testicular Cells

M2A-positive and -negative populations were sorted from human fetal testes that had been exposed or not to 10^−4^ M MEHP for three days and the mRNA expression of the three NRs was analyzed in both fractions (Figure 5A). *PPARγ* was detected only in M2A-positive cells and was not statistically modified in comparison to untreated controls. *NGFIB* expression was down-regulated in M2A-positive cells (−30% in comparison to untreated controls) and a trend to diminution was observed in M2A-negative cells. *LXRα* expression was significantly up-regulated following MEHP exposure in M2A-negative cells (45% increase), and unchanged in M2A-positive cells (Figure 5A).

As *LXRα* transcriptional up-regulation was restricted to somatic cells, we investigated whether *in vitro* exposure to MEHP affected the lipid and cholesterol synthesis pathways only in M2A-negative cells. Indeed, the six genes we found to be up-regulated in whole testes (Figure 2A) showed a 2- to 3-times significant increase only in M2A- negative cells, whereas in M2A-positive cells their expression was comparable to that of untreated controls (Figure 5B).

### Physiological Consequences of MEHP Exposure on Lipids Synthesis in the Testicular Somatic Cells

To test if the transcriptional regulation observed after MEHP exposure really affects lipid synthesis in the testicular somatic cells, we performed analyses of lipid vesicle number per nuclei and average area after MEHP exposure (10^−4^ M) during 72 h. Briefly, human testicular cells were dispersed and Oil Red O staining was performed to reveal the lipidic vesicles. Since MEHP effect on lipidic pathway was observed only in somatic cells, M2A immunological staining was performed to identify and exclude germ cells. Appearance of the lipidic vesicles is presented in figure 6A. The treatment with 10^−4^ M MEHP significantly increases the average vesicle area in the somatic cells without modifying the number of vesicle per nuclei (Figure 6B).

## Discussion

The main purpose of this work was to broaden our knowledge regarding novel effects of phthalates exposure on human fetal gonads during pregnancy. Several studies have highlighted the potential involvement of different NR in the mediation of phthalate effects.

To realize this study, we set up a new protocol to sort for the first time human male fetal germ cells by a cytometric approach. This sorting strategy, using M2A antigen expressed in 90 to 95% of the germ cell population during the first trimester of pregnancy, will allow studies of human fetal male germ cells, a very important issue as the gonocytes are the precursors of the adult germ stem cells and are supposed to be the cells whom testicular cancer arise [43].

In order to determine to what extent NRs are involved in the testis response to phthalate exposure, we analyzed, using TLDA plates, the effect of a 3-day *in vitro* MEHP treatment on the mRNA expression of the 48 known NRs in human fetal testes. In basal conditions, Chicken Ovalbumin Upstream Promoter-Transcription Factor II *(COUP-TFII*), a NR that is mainly expressed in Leydig cells and is involved in testosterone synthesis in mouse fetal testes [44], showed the highest expression. This result is not surprising as we used first-trimester human fetal testes, in which the production of testosterone and the number of Leydig cells rise to their maximum [45]. Interestingly, the Orphan Nuclear Receptor Small Heterodimer Partner (*NR0B2*) that in mouse neonatal testis mediates the deleterious effects of DES, a well-known endocrine disruptor [46], was not detectable in human fetal testes (Table S2).

*PPARγ* expression was significantly decreased in whole testes and we determined that its expression is restricted to the germ cell in male. Therefore, the observed decrease in *PPARγ* mRNA probably reflects the loss of gonocytes induced by MEHP exposure in the cultured human testis [16]. Concerning the decrease in *NGFIB* mRNA expression, transcriptomic analyses performed in rodent fetal testes have revealed that *in vivo* exposure to different phthalates increases the mRNA expression of *NGFIB* and *NOR1*, [21], [32], [33]. On the contrary we observed here a decrease in the human fetal testis and in fetal germ cells, exhibiting here a new specificity of the human species.

We also demonstrated here that *LXRα* mRNA was up-regulated both in male and female fetal gonads. This is the first demonstration that *LXRα* mRNA expression is modulated by phthalate exposure. Indeed, LXRα was never incriminated in the literature whatever the species or the tissues analyzed. Particularly, the global transcriptomic analyses performed using rat or mouse fetal testes after exposure to phthalates [21], [33] failed to identify *LXRα* among the differentially expressed genes.

We then show that the cholesterol and lipid synthesis pathways, known to be regulated by LXRα through SREBP members [47], [48] are also up-regulated in response to MEHP exposure in male human fetal gonads. Moreover, the increased expression of the lipid/cholesterol pathways is correlated to an increase in the total amount of lipids produced in the somatic cell of the human testis. This result fits with the well-known control of the LXR receptors over the lipid homeostasis. Indeed, in the literature, effects of phthalates modulating metabolic processes and particularly cholesterol and lipid synthesis have already been described. *In utero* DEHP exposure alters post-natal liver development in mice causing significant increased hepatosteatosis [49]. In the same way, it has already been reported that in adipocytes differentiated *in vitro* MEHP exposure increases the triglyceride contents [50].

A recent study described a decrease in the expression of *SREBP2* and downstream genes in fetal rat testis following *in utero* exposure to phthalates, resulting in reduced cholesterol level in whole testes [51]. The authors linked this result to the well-known effect of phthalates on testosterone production. Conversely, in our model of human fetal testis culture, such a link between cholesterol synthesis and testosterone secretion is not so obvious, as testosterone production was never modified by MEHP treatment [16]. This might reflect species-specific effects, as, in the same study, Johnson and collaborators reported that *in utero* exposure to phthalates in mice increased the expression of *SREBP2* and downstream genes without changes in testosterone production [51].

Despite this classical role, LXRs are also known to be involved in various physiological processes such as: glucose homeostasis, immunity and steroidogenesis [52]. LXR is also described to act in both female and male reproductive tissues [53], [54]. In mice testis lacking LXRα, it has been evidenced a higher accumulation of the proapoptotic transcripts Bad and TNF-α associated with a higher number of apoptotic cells [55]. In the same way, LXR-deficient mice present abnormal epididymal features [53]. Lastly, LXRα controls the proliferation versus apoptosis balance in prostate cancer cells [53]. In the female side, LXRα modulates steroids synthesis in mice ovaries. In placenta, LXRα is known to regulate cholesterol transport and trophoblastic invasion [54]. It will be essential to further investigate if these LXR-related processes are altered in our model of human fetal gonads.

*LXRα* expression was increased in treated fetal ovaries. This not only demonstrates that human fetal ovary is sensitive to phthalate exposure, but also highlights a similar response to MEHP treatment in male and female human fetal gonads. On the other hand, the germ cells loss exclusively restricted to the male side suggests that some (but not all) phthalate effects are sex-specific [16]. This correlate with previous data demonstrating that human male and female germ cells can respond differently to external stress signals [56]. In the same way, *in utero* DEHP exposure in rat showed opposite effects on aldosterone levels in male and female at adulthood [57]. It could be interesting to analyze whether MEHP can have other effects on female gonads. A recent study shows that female adult mice treated with phthalate during their embryonic life have an elevated estradiol level and altered mRNA expression was found for the steroidogenic genes Luteinizing Hormone/Choriogonadotropin Receptor *(LHCGR)*, aromatase *(CYP19A1)*, and *StAR* [58].

We evidenced that the lipid/cholesterol pathways is altered specifically in the somatic cell population of the human testes and that germ cells loss is also exclusively restricted to the fetal testis. So far, we cannot conclude to an interaction between this altered lipid/cholesterol pathway in somatic cells and the germ cell loss we previously evidenced [16], [17]. However, as somatic cells are known to impact germ cells proliferation and apoptosis, such a relation cannot be excluded and further investigation using LXRα antagonist or gene silencing technology will be required.

To conclude, this work evidences for the first time a novel effect of phthalate exposure involving up-regulation of *LXRα* and *SREBP* members mRNA that control both cholesterol and lipid synthesis (Figure 7). Thanks to our new cell sorting approach, we demonstrated that this effect is restricted to the somatic cells in the testis. Moreover, this up-regulation is observable in both human male and female fetal gonads in our *in vitro* culture system. However, these results do not establish which relevant physiological consequences this novel phthalate effect may trigger in the developing gonads.

## Supporting Information

Table S1 **List of the different TaqMan and SYBR primers used in qPCR along this study.**
(DOCX)

Table S2 **List of the nuclear receptors expressed in the human fetal testis.** All mRNA expressions were normalized on Actin β. The lowest mean ΔCt value refers to the highest expressed nuclear receptor.
(DOCX)
