# Supplementary material for: Cellular and Molecular Effect of MEHP Involving LXRα in Human Fetal Testis and Ovary
Source: PLoS One. 2012 Oct 30;7(10):e48266. doi: 10.1371/journal.pone.0048266 (PMC3484128; doi:10.1371/journal.pone.0048266)
Supplement: Table S1 — List of the different TaqMan and SYBR primers used in qPCR along this study. (DOCX) [file pone.0048266.s001.docx]

***Table S1:*** List of the different TaqMan and SYBR primers used in qPCR along this study*.*

| **TaqMan Primers** | | **SYBR Primers** | |  |  |  |
| --- | --- | --- | --- | --- | --- | --- |
| Genes | Reference | Genes | Forward sequence | | Reverse sequence |  |
| *AMH* | Hs00174915_m1 | *SCD1* | CATAACAGCAGGAGCTCATCGT | | ACGAGCCCATTCATAGACATCA | |
| *StAR* | Hs00264912_m1 | *SERBP1c* | TGCAACACAGCAACCAGAAA | | GAGATTTGCTTTTGTGGACAG | |
| *NR4A1* | Hs00378230_m1 | *HMGCoARed* | GATGGGAGGCCACAAAGAG | | TTCGGTGGCCTCTAGTGAGA | |
| *PPARγ* | Hs00234592_m1 | *SqEp* | CAACAGTCATTCCTCCACCA | | AGCAAGCTTCCTTCCTCCTT | |
| *LXRα* | Hs00172885_m1 | *VASA* | GCCTCTGGGCGGAATTTT | | CGCTTATTACACTCACCAGCATC | |
| *RPLP0* | Hs99999902_m1 | *M2A* | TGTGGTTATGCGAAAAATGTCG | | CCTTCAGCTCTTTAGGGCGAG | |
| *Actin β* | Hs99999903_m1 | *FASN* | GCTCCAGCCTCGCTCTC  GAGACCATGGAGACCCTCAC | | TCTCCGACTCTGGCAGCTT  TCAGGGAACTCTCCCACTTG | |
|  |  | *SREBP2* |  |  |  |  |
