# Supplementary material for: Cellular and Molecular Effect of MEHP Involving LXRα in Human Fetal Testis and Ovary
Source: PLoS One. 2012 Oct 30;7(10):e48266. doi: 10.1371/journal.pone.0048266 (PMC3484128; doi:10.1371/journal.pone.0048266)
Supplement: Table S2 — List of the nuclear receptors expressed in the human fetal testis. All mRNA expressions were normalized on Actin β. The lowest mean ΔCt value refers to the highest expressed nuclear receptor. (DOCX) [file pone.0048266.s002.docx]

***Table S2:* List of the nuclear receptors expressed in the human fetal testis.** All mRNA expressions were normalized on Actin β. The lowest mean ∆Ct value refers to the highest expressed nuclear receptor.

| *NUCLEAR RECEPTOR* | | | | | | |  |  |
| --- | --- | --- | --- | --- | --- | --- | --- | --- |
| **Member** | **Classification** | **Full name** | | | | | **Mean** ∆Ct | **St. Dev.** |
| *COUP-TFII* | NR2F2 | Chicken Ovalbumin Upstream Promoter-Transcription Factor II | | | | | 2,36 | 0,3 |
| *EAR-2* | NR2F6 | V-erbA-related protein 2 | | | | | 4,03 | 0,28 |
| *RXRβ* | NR2B2 | Retinoid X Receptor beta | | | | | 4,08 | 0,37 |
| *SF1* | NR5A1 | Steroidogenic Factor 1 | | | | | 4,16 | 0,51 |
| *LXRβ* | NR1H2 | Liver X Receptor beta | | | | | 4,67 | 0,33 |
| *RXRα* | NR2B1 | Retinoid X Receptor alpha | | | | | 4,83 | 0,27 |
| *GR* | NR3C1 | Glucocorticoïd Receptor | | | | | 4,89 | 0,22 |
| *PPARβ/δ* | NR1C2 | Peroxisome Proliferator-Activated Receptor delta | | | | | 5,16 | 0,33 |
| *TR4* | NR2C2 | Testicular Receptor 4 | | | | | 5,2 | 0,26 |
| *TRα* | NR1A1 | Thyroid hormone Receptor alpha | | | | | 5,42 | 0,35 |
| *Rev-ErbAβ* | NR1D2 | Reverse ErbA beta | | | | | 5,76 | 0,39 |
| *RARα* | NR1B1 | Retinoic Acid Receptor alpha | | | | | 5,98 | 0,16 |
| *ERRα* | NR3B1 | Estrogen receptor-Related Receptor alpha | | | | | 5,99 | 0,61 |
| *COUP-TFI* | NR2F1 | Chicken Ovalbumin Upstream Promoter-Transcription Factor I | | | | | 6,32 | 0,6 |
| *TR2* | NR2C1 | Testicular Receptor 2 | | | | | 6,46 | 0,36 |
| *NGFIB* | NR4A1 | Nerve Growth Factor-Induced factor B | | | | | 6,6 | 0,45 |
| *GCNF* | NR6A1 | Germ Cell Nuclear Factor |  |  |  |  | 6,63 | 0,9 |
| *DAX1* | NR0B1 | Dosage-sensitive sex reversal, Adrenal hypoplasia critical region on X chr gene 1 | | | | | 6,67 | 0,77 |
| *Erβ* | NR3A2 | Estrogen Receptor beta |  |  |  |  | 7,44 | 0,43 |
| *LXRα* | NR1H3 | Liver X Receptor alpha |  |  |  |  | 7,72 | 0,21 |
| *RARβ* | NR1B2 | Retinoic Acid Receptor beta |  |  |  |  | 7,73 | 0,64 |
| *HNF4α* | NR2A1 | Hepatocyte Nuclear Factor 4-alpha | |  |  |  | 7,84 | 0,52 |
| *AR* | NR3C4 | Androgen Receptor |  |  |  |  | 7,88 | 0,42 |
| *Rev-ErbAα* | NR1D1 | Reverse ErbA alpha |  |  |  |  | 7,9 | 0,24 |
| *Erα* | NR3A1 | Estrogen Receptor alpha |  |  |  |  | 8,59 | 0,25 |
| *RORα* | NR1F1 | RAR-related Orphan Receptor alpha | |  |  |  | 8,73 | 0,32 |
| *VDR* | NR1I1 | Vitamin D Receptor |  |  |  |  | 8,73 | 0,32 |
| *PPARγ* | NR1C3 | Peroxisome Proliferator-Activated Receptor gamma | | |  |  | 8,93 | 0,28 |
| *PPARα* | NR1C1 | Peroxisome Proliferator-Activated Receptor alpha | | |  |  | 9 | 0,36 |
| *NOR1* | NR4A3 | Neuron-derived Orphan Receptor 1 | |  |  |  | 9,23 | 0,52 |
| *RARγ* | NR1B3 | Retinoic Acid Receptor gamma |  |  |  |  | 9,3 | 0,5 |
| *LRH-1* | NR5A2 | Liver Receptor Homologous protein 1 | |  |  |  | 9,54 | 0,92 |
| *RORβ* | NR1F2 | RAR-related Orphan Receptor beta | |  |  |  | 9,59 | 0,69 |
| *NURR1* | NR4A2 | NUR Related factor 1 | | | | | 9,85 | 0,23 |
| *TRβ* | NR1A2 | Thyroid hormone Receptor beta | |  |  |  | 10,01 | 0,49 |
| *MR* | NR3C2 | Mineralocorticoïd Receptor |  |  |  |  | 10,13 | 0,62 |
| *RXRγ* | NR2B3 | Retinoid X Receptor gamma |  |  |  |  | 10,45 | 1,19 |
| *FXR* | NR1H4 | Farnesoid X Receptor |  |  |  |  | 10,89 | 0,53 |
| *RORγ* | NR1F3 | RAR-related Orphan Receptor gamma | |  |  |  | 11,56 | 0,63 |
| *ERRγ* | NR3B3 | Estrogen receptor-Related Receptor γ | | | | | 11,68 | 0,5 |
| *PXR* | NR1I2 | Pregnan X Receptor |  |  |  |  | 12,33 | 0,39 |
